# Annotations

**Published:** 1900-02-03

**Authors:** 


### ANNOTATIONS.

### Leaky Water Pipes.

WHENEVER the town happens to be short of water, or when, as happens now and again, an explosion occurs in an electric light box, we hear much about the leaky conditions of the gas mains and the water pipes which honeycomb the ground beneath every street. At other times we conveniently forget all about it. Nevertheless, it goes on all the while, and whether we think of it or not it remains a constant danger and cause of ill-health. Let us confine our attention to water alone. It has been stated by water engineers who have devoted much attention to the subject that as much water is lost by leakage, and in many cases more than is supplied to the consumers. Some years ago Sir Frederick Bramwell presented to the British Institution of Civil Engineers a diagram showing the relation between the amount pumped into the mains and the amount actually used in certain towns in England where meters were in use, and by its means he showed that about twice as much was lost as was accounted for. A still worse condition has lately been shown to exist in Philadelphia. In that city, where, according to the *Scientific American*, a daily consumption of 523 gallons per head of population is recorded, the total day consumption only exceeds the night consumption by about 20 per cent. Who is using this vast volume of water all the night through? The conclusion is obvious, that for every gallon that is used four or five gallons are lost by leakage. Let us apply this to ourselves. The first essentials to a healthy home are a dry sub-soil and a pure ground air. Yet all the water companies and all the gas companies are engaged, year in, year out, in soaking the sub-soil with water and fouling the ground-air with gas, much of which, owing to its almost constant admixture with water-gas is definitely poisonous. A still more serious effect of leaky water-pipes arises from the fact that when clean water can get out, dirty water can get in. Running side by side with drains and sewers, and only a short distance below manure-clad streets, which in many cases are freely open to percolation, lie the leaky pipes conveying water which, as we are constantly told, is "well filtered and of a high degree of purity." But when the turncock goes his rounds and turns off the supply, then these pipes are quickly emptied and become little else but drains. The puddle in which they lie, caused by their leakage, is now sucked in again carrying with it the washings of the subsoil. And this we drink. When, then, we hear it suggested that London should purchase, among other things, the pipes of the water companies, it may be well to point out that the question of leakage has a very different bearing according as one is a vendor of the water or has to drink it. So far as dividends are concerned, it may be cheaper to pump more water than to mend the leaks; but leaky water-mains are a danger to those who have to drink their contents.

### Municipal Hospitals.

IN an article which appeared lately in the *National Review* Miss Honnor Morten attempts to show how great would be the advantages of municipalising the hospitals of this country. She maintains that among the great disadvantages of the voluntary system is "the corruption due to a system of charity," and that "mercy may bless both him that gives and him that takes, but charity turns the first into a complacent humbug, and the second into a degraded hypocrite." She also say that "it is obvious that in all other countries there is greater public control of hospitals than we have in England, and that the result is a more free and sensible use of the advantages of hospital treatment." For her it would seem that all that is Continental or American is superior to what is English, and that our hospitals are especially bad in that the doctors are uncontrolled. To show what desperate deeds are done by doctors uncontrolled she quotes from THE HOSPITAL of December 24th, 1898, a series of cases of tumours, cancerous and otherwise, treated by toxins and sera, or, as she elegantly puts it, by "injecting the stale blood of diseased horses," all of which will no doubt be accepted as peculiarly apropos by her innocent readers, who will take it as proof positive that, as she says, "such experimenters ought to be under some form of control." Poor Miss Morten, however, seems to have neglected the very obvious precaution of inquiring where these terrible deeds were done. If she had gone further she would have found that it is from her beloved America and Germany and France, where things are managed so much better than in England, that these tales come!

### Men in Armour.

IN view of the brave doings of the gentlemen in khaki ordered South, a dash of the ridiculous attaches to the notion of going into battle clad in armour. Recent experience of the effects of modern bullets and the results obtained by modern surgery have, however, brought up again the old idea although in a new form. The extraordinary recoveries which have occurred show that, if an absolutely vital part be not struck, there is a fair chance of recovery wherever a man is hit, and, under these circumstances it is not unnatural to jump at the suggestion that if only these vital parts could be protected all might be well. Mr. Jonathan Hutchinson[1] says that never before in the history of the world were wounded men more rapidly restored to the combatant ranks, and he points to the different meaning the term "wounded" comes to have when two-thirds of the men coming under that head may probably be back in the ranks within a fortnight. Now that, as he says, to be shot through the lungs involves but little risk, and that bullet wounds of the limbs count for almost nothing, whilst those of the abdomen, the liver, and even of the head are far from being necessarily fatal, the region of the heart remains the fatally vulnerable part, and he suggests that it may perhaps be found practicable to contrive a breast-plate which may, without undue encumbrance, conduce much to the wearer's security. The idea is good, and perhaps if the so-called "frontal attacks," which have cost us so much, are to remain in fashion we may come to it. What, however, does not seem to have been taken note of by those who suggest the wearing of the breast-plate is that bullets in modern warfare do not always, perhaps do not even generally, hit a man in front. With the "stalking" tactics which are now essential a breast-plate, however perfect, would only exceptionally be of service. Nothing that adds to the load carried by a foot soldier can do anything but add to his danger. It is more than ever becoming clear that the soldier's safety lies in rapid movement, not in making him either a beast of burden or even a man in armour.

[1] Polyclinic, January, 1900.

